# Supplementary material for: Are carbon emissions trading and green financial instruments synergistic? -Comprehensive quantitative research based on content analysis
Source: PLoS One. 2024 Mar 7;19(3):e0298601. doi: 10.1371/journal.pone.0298601 (PMC10919601; doi:10.1371/journal.pone.0298601)
Supplement: S1 Data — (ZIP) [file pone.0298601.s001.zip › Data/地级市Z_F工作报告.zip!/║┌┴·╜¡/╝╤─╛╦╣╩╨/└┤╫╘╣1⁄2╓┌║┼í╛┬φ┐╦╩2╛▌═°í┐_2017.docx]

各位代表：

现在，我代表市人民政府向大会报告工作，请予审议，并请市政协委员提出意见。

一、过去五年及2016年工作回顾

过去的五年，是我市综合实力提升最快、转型发展进展最大、人民群众得到实惠最多的时期之一。五年来，面对复杂的宏观经济环境和艰巨的改革发展任务，我们以习近平总书记系列重要讲话精神，特别是对我省两次重要讲话精神为根本遵循，紧扣省委、省政府和市委的各项决策部署，坚持一张蓝图绘到底，一以贯之抓落实，较好地完成了既定目标任务。

五年来，我们聚焦科学发展，持续提升综合实力。遵循发展规律，系统谋划全局，在完成“十二五”规划目标基础上，科学编制并启动实施了“十三五”规划，预计国内生产总值比2011年增长63.6%，固定资产投资增加1.2倍，发展基础更加坚实。

五年来，我们立足结构调整，加快推进产业升级。发挥自身优势，抢抓政策机遇，开复工5000万元以上产业项目783个，竣工投产550个，一批补短板、增后劲的重大项目成为拉动产业升级的重要引擎，初步构建起具有较强竞争力和支撑力的现代产业新格局，发展后劲持续增强。

五年来，我们突出城市定位，不断扩大辐射影响。着力打造商贸物流、教育医疗、休闲文化、科技金融、立体综合交通“五个中心”，建成国家级园林城市，实现全国双拥模范城“八连冠”，综合承载功能日趋完善，整体环境宜居宜业，城市能级加快提升。

五年来，我们坚持需求导向，全力办好民生实事。注重民生工作的持续性、渐进性、积累性，累计投入民生资金621.1亿元，占公共财政预算支出的63.4%，全面启动脱贫攻坚，预计城乡居民人均可支配收入比2011年分别增长61%和54%；城乡低保标准分别提高6%和6.3%，筑牢了民生改善之基、社会转型之实。

五年来，我们全面从严治政，依法规范政府管理。注重管理创新，完善行政决策合法性审查机制，发挥土地管理委员会、规划专家评审委员会等议事机构法定职能，理顺相关议程，扎紧制度笼子；“放管服”改革省内先行，政府工作的规范性、系统性、实效性不断增强。

刚刚过去的2016年，是本届政府的收官之年。我们在省委、省政府和市委的坚强领导下，准确把握战略机遇期内涵的深刻变化，在规范调整中进取，在破解难题中前行，全市经济社会发展稳中有进，实现了“十三五”良好开局。预计地区生产总值增长6.5%左右；固定资产投资完成587亿元，增长10%左右；社会消费品零售总额实现445亿元，增长11%左右。一般预算收入完成37.7亿元，增长4.7%。城乡居民人均可支配收入增长与经济增长同步。

（一）在扩大有效投资中强化项目建设。开复工产业项目903项，完成投资410亿元，同比增长10.9%，占固定资产投资的70%；竣工投产项目600个以上。产业项目开复工数量、投资增幅及省重点项目开复工率等指标均位居全省前列。泉林项目一期实现试生产，象屿集团200万吨玉米深加工项目一期正式投产，万里利达粮食仓储及深加工项目当年开工建设、当年启动收储。牡佳客专开工，哈佳快速铁路市辖区内施工进度全线领先，佳木斯站综合改造等重大基础设施项目顺利推进，38个国省水利项目超额完成年度投资计划。依托各类有效载体开展产业招商，形成有价值的项目线索108个，有60个项目签订合同并履约，招商引资到位资金同比增长15%。各级园区的项目承载和产业集聚功能进一步增强。

（二）在加快转变方式中发展现代农业。新增优质水稻面积10.5万亩、大豆面积51.1万亩，调减玉米面积76万亩；以亮子奔腾等龙头企业为牵动，食用菌栽培3.5亿袋以上。推进畜牧业产业化、规模化、标准化和生态化建设，双兆猪业300万头生猪养殖项目实现开工，汤原盛世凯达奶牛场项目进展顺利。落实万亩以上“三减”示范基地83处，开辟黄腐酸有机肥示范点152处，完成测土配方施肥面积803万亩，全省农业“三减”现场会在我市召开。建成“互联网+农业”高标准示范基地102处，鱼鸭蟹稻等有机种植面积发展到3.7万亩。新增农民合作社214个，各类新型经营主体辐射农户超过10万户。流转土地面积占集体耕地的68.5%，农村土地确权登记达到待发证状态。探索推行14种新型农贷业务，贷款利率稳中有降。新组建现代农机合作社18个，农机调度指挥中心建成运行。实施新城等4个灌区项目，新增和恢复灌溉面积1.86万亩，改善灌溉面积7.94万亩，建设高标准农田4.29万亩。组织“品牌农业中国行·佳木斯大米走进大连”等整体营销活动12次，金玛集团等龙头企业、桦川五良纯等专业合作社通过自建或“互联网+”平台开展直销，助推佳木斯大米等优质农产品由“种的好”向“卖的好”转变。

（三）在努力做大增量中优化工业结构。开复工5000万元以上重点工业项目36个，佳星玻璃综合技改、润特科技特种电缆生产线等企业改造项目开工建设，传统产业改造升级和存量创新步伐加快。积极争取政策支持，为各类企业落实贷款20.6亿元。推进企业梯次成长升级，新增企业4363户，新进规模以上工业企业8户。成立科技型企业177户，新增一定规模科技型企业37户，转化科技成果71项，交易额超过2100万元；新建孵化器和创客空间10个，新增入孵企业和项目146个；新增国家高新技术企业4户、省级专利优势试点企业3户。

（四）在促进消费升级中释放需求空间。万达广场室外步行街等项目有序建设，利达双子星奥莱正式运营。75户企业入驻佳木斯电子商务产业园及创客空间。有序整合以民族文化游为重点的特色旅游产业，推出沿松花江到黑龙江的少数民族特色生态黄金旅游线路，同江三江口生态旅游区成功晋升国家4A级旅游景区。全市旅游总收入突破19亿元。

与15家外省养老机构签订意向候鸟养老协议和投资意向性合作协议，规划候鸟旅游路线3条，建设医养结合养老机构10家。中投证券、中信银行在我市设立分支机构，农发行黑龙江省分行、农行黑龙江省分行、龙江银行与我市签订战略合作协议。17家企业纳入省拟上市企业后备资源库，3户企业报“新三板”待批。

（五）在推进改革开放中激发市场活力。深化“放管服”改革，建立行政权力清单、责任清单和行政职权运行流程的动态管理机制，初步形成行政权力中介服务清单，制定涉企收费清单。市直27个部门向县（市）区下放行政职权1041项，基层政府工作的主动性、积极性明显增强。推行“互联网+政务服务”，市级35个单位433项行政审批类事项全部编录网上行政审批系统。市公共资源交易平台完成整合，国有资产、政府采购等项目统一进入中心公开交易。实施“五证合一、一照一码”和“两证整合”登记制度改革，全市新增各类市场主体24989个。试行“营改增”，为企业减负7亿元。启动跨年度预算平衡机制，财政资金支付实现全程动态监控。城管综合执法体制改革工作在全国城市管理培训班上做经验介绍。供销社等改革工作稳步推进。同江中俄跨江铁路大桥中方工程即将进入初步验收阶段，佳木斯至比罗比詹运输线路延伸至哈巴罗夫斯克，佳木斯空港货运通道、江海联运开通等事宜正在协商推进。规划完成佳木斯综合保税区、同江跨境经济合作区等合作开放平台，佳天国际农副产品批发中心海关监管库建成使用。

（六）在突出惠民利民中增进民生福祉。全市民生投入157亿元，占公共预算支出的68.2%。推进脱贫攻坚，完成脱贫任务6.3万人。完善创业平台，培育扶持525名大学生、1633名农民工成功创业。城镇新增就业4.8万人，登记失业率控制在4.08%以内。城乡“五险”实现扩面。新建续建城市道路12条，学府街、胜利路两座立交桥简易通车。推进城区热源项目3项，新增供热能力525万平方米；新建改造燃气管网74公里，新增燃气用户14568户。建立长效管理机制，城市“三乱”现象得到有效遏制。采购大型清雪设备70台套，实现城区主街、巷道同步机械化清雪作业。完成王三五河等内河整治工作，松花江干流佳木斯江段Ⅲ类水质比例100%。全年建成美丽乡村120个。解决13个村屯1.05万人口饮水安全问题。改造棚户区11778套、85.59万平方米，超额完成省计划任务。改造农村危（草）房10624户。按计划开工建设保障性安居工程和公租房，新增廉租房租金补贴700户。公积金归集额、提取额分别增长14.3%、11.2%。维修城区老旧楼房443栋。佳西客运站竣工，33台新能源纯电动公交车上线运营。推进义务教育优质均衡发展，中小学校标准化建设实现100%。各县（市）13家公立医院取消药品加成，全面实施12类46项基本公共卫生服务。在全省率先实行新农合异地就医即时报结、建立第三方医疗纠纷调解模式。创作《乌苏里传歌》等一批文艺精品，组织开展“百团大赛”等群众文化系列活动。以佳木斯快乐舞步为代表的群众体育蓬勃发展，被评为省内唯一“全民健身城”。新增规范化社区用房40个，创建省级“双百”示范社区34个。连续五年入选“中国十佳食品安全城市”，被国务院食安办批准为全省首批创建国家食品安全城试点，自创纪录片《舌尖上的佳木斯》在央视交通频道播出。畅通社会矛盾导入司法渠道，群众来信、来访、来电同比下降20.4%。安全生产形势总体稳定，社会治安状况群众满意度持续提高，市公安局获得“全国公安机关执法示范单位”荣誉。

（七）在推进管理创新中加强自身建设。成立市政府专家咨询顾问委员会，完善科学民主决策机制。自觉接受各方监督，人大代表建议、政协委员提案答复率100%。发挥政务微博、微信及手机APP平台作用，拓展政民互动空间，在全省率先开展“听民意、聚民智、惠民生”问政活动。应急指挥平台正式投入运行。加大对影响制约科学发展、损害群众利益等问题的追究问责力度，发展环境得到优化。严格开展行政监察和审计监督，市本级“三公”经费同比下降17.3%。

外事侨务、民族宗教、机关事务、质监、统计、气象、地震、人防、接待、档案等工作得到加强，妇女儿童、残联、红十字等工作扎实推进，中省直部门为地方发展做出突出贡献。

各位代表，我们取得的成绩来之不易，这是省委、省政府和市委正确领导的结果，是全市上下共同努力、社会各界鼎力支持的结果。在此，我代表市人民政府，向各位代表和全市人民，向离退休老领导、老同志，向民主党派、工商联、人民团体，向各位企业家、科技工作者，向驻佳部队和武警官兵，向各中省直单位，向所有关心支持佳木斯发展的各界人士、国际友人和海内外佳木斯籍人，致以崇高的敬意和衷心的感谢！

在总结成绩的同时，我们也必须正视当前发展面临的突出问题。主要是产业缺乏市场竞争力，主导产业支撑力不强、新兴产业发展滞后；民生欠帐较多，一些群众关心的热点难点问题没有完全得到解决；发展环境有待进一步改善，有的干部思想理念陈旧、专业化水平不高，个别干部不担当、不作为现象仍然存在等。我们必须以担当有为的精神和改革创新的勇气，集思广益积极应对，持续发力加以解决。

二、今后五年总体思路及2017年工作安排

今后五年，是佳木斯决胜全面小康、推动振兴发展的关键机遇期。政府工作的指导思想是：高举中国特色社会主义伟大旗帜，以邓小平理论、“三个代表”重要思想和科学发展观为指导，深入贯彻习近平总书记系列重要讲话精神，特别是持续贯彻落实对我省两次重要讲话精神，紧紧围绕中央“五位一体”总体布局、“四个全面”战略布局，牢固树立新发展理念，贯彻稳中求进工作总基调，按照省委、省政府和市委的总体要求，以赶超的勇气谋发展，以务实的精神补短板，以创新的思维促改革，坚持“五个工作导向”，实施“四大升级工程”，推进“五项建设任务”，不断壮大经济实力，优化产业结构，增进民生福祉，为决胜全面小康，实现强市富民而努力奋斗。

未来五年的奋斗目标是：地区生产总值年均增长6.5%左右，全社会固定资产投资年均增长10%左右，一般公共财政预算收入年均增长6%左右，规模以上工业增加值年均增长10%左右，社会消费品零售总额年均增长10%左右，城乡居民人均可支配收入增长与经济增长同步。

以上目标，是立足实际和可能，衔接全面建成小康社会和“十三五”规划的各项指标，经过反复比较、综合权衡后确定的，既保证经济运行在合理区间，又保障供给侧结构性改革、民生改善和财政增长，为增强社会和市场信心创造条件。我们要立足“五大发展优势”，抢抓“四大发展机遇”，着力构建“四大体系”，确保完成既定任务。

——构建现代产业体系。做强“四大支柱产业”，做优“三大战略性新兴产业”，培育“五大特色和潜力产业”，推动产业层次向中高端发展，创建老工业基地产业转型升级先导区。以推进农业供给侧结构性改革为主线，以品牌农业建设为引领，以建设国家绿色食品生产样板区、高效生态农业先行区为载体，优化农业产业体系、生产体系、经营体系，加快建立绿色食品全产业链生态循环体系，争当农业现代化建设排头兵。加快各类要素资本集聚，推动生活服务业向精细化和高品质转变、生产服务业向专业化和价值链高端延伸，全力打造现代服务业升级版。深入发掘旅游文化资源市场化潜力，培育特色线路，做大旅游经济。

——构建新型开放体系。积极对接“一带一路”和“龙江丝路带”战略，完善大通道，打造大平台，集聚大产业。依托同江铁路大桥两端产业和贸易互补，建设承接国内外产业转移示范区和对外贸易加工聚集带，促进境内外产业联动、上下游产业衔接，打造“龙江丝路带”重要枢纽和窗口城市。落实国务院关于建立东南沿海与东北对口合作机制的决策部署，强化与国内先进地区的交流合作和产业互动，持续释放我市各类优势资源活力。

——构建绿色生态体系。树牢绿水青山就是金山银山的理念，争创国家生态文明试验区。强化黑土地和松花江、黑龙江生态资源的保护与治理，创造良好的生态安全环境，以环境保护转变发展方式、拓展发展空间，让良好生态成为可持续发展的竞争优势。推进资源集约利用，形成以循环经济、绿色食品、林下经济、健康养老为主体的绿色生态产业体系，建设绿色振兴、转型升级示范区。

——构建民生保障体系。把握“坚守底线、突出重点、完善制度、引导预期”的基本思路，顺应群众对美好生活的新期盼，找准民生改善与经济发展的结合点，不断完善就业、教育、文化、社保、医疗、住房等公共服务体系，补齐民生领域短板，提前完成脱贫攻坚任务，全面建成小康社会，使全市人民更公平地分享发展成果。

各位代表，2017年是新一届政府施政的开局之年，也是实施“十三五”规划的重要一年。我们要自觉将思想和行动统一到省委、省政府和市委对当前形势的综合研判、对全面工作的既定安排上来，深入贯彻落实市十三次党代会精神，以深化供给侧结构性改革为牵动，持续推进“三去一降一补”，牢牢把握加快科学发展的主动权。

全市经济社会发展的主要预期目标是：地区生产总值增长6.5%左右；全社会固定资产投资增长10%左右；社会消费品零售总额增长10%左右；一般公共预算收入增长3%左右。城乡居民人均可支配收入增长与经济增长同步。

为实现上述目标，着力抓好六个方面工作：

（一）促进投资精准有效。围绕实施“四大升级工程”，构建“四个体系”谋划推进项目，引领项目建设工作重心向“稳、准、实”聚焦发力。

加快哈佳快速铁路、牡佳客专、佳木斯站综合改造项目建设，确保投资和进度，实现公铁联运客运枢纽站等项目落地开工，推进机场迁建、松花江二桥等项目取得积极进展。开复工产业项目900项，产业项目投资增长10%左右。重点推进泉林秸秆综合加工二期等项目，以秸秆资源原料化、肥料化、饲料化、基料化利用为方向，延伸综合利用产业链，发展循环经济。加快万里利达粮食仓储及深加工等项目建设，实现300个以上项目竣工投产。开展科学化、专业化、差异化招商，招商引资到位资金同比增长15%以上，力争在引进龙头、扬长补短上取得突破。完善园区基础设施，推动公共服务体系集约化运行。探索“异地共建”“税收分享”等合作模式，提高园区土地利用强度和投入产出效益，推进佳木斯高新区晋升国家级开发区申报工作。

（二）加快产业转型升级。把转方式调结构作为推动发展的重中之重，抢抓“四大机遇”，坚定不移做好“三篇大文章”。

聚力现代农业。以提高农业供给质量为方向，培育农业农村发展新动能，提高农业综合效益和竞争力。加快现代农业生产体系建设。推进引汤灌区等农田水利工程新建、续建，新增和改善农田灌溉面积5.2万亩以上，规划建设生态高标准农田32万亩，稳定提高粮食综合生产能力。启动新一轮农网改造升级，实施农村井井通电工程。新建农机合作社10个以上，推动农机装备结构优化升级。科学实施农业“三减”，全市化肥农药除草剂分别减量8%、10%、20%以上。推广使用黄腐酸肥，促进土壤有机质含量恢复性增长。培育发展现代种业，建设北方寒地水稻种子交易中心和东北水稻种业基地。加快现代农业产业体系建设。新增水稻、大豆、蔬菜种植面积20万亩、50万亩、10万亩以上，食用菌发展到4亿袋以上，玉米调减到700万亩以下，稳步扩大红树莓、紫苏等特色作物种植规模。“互联网+农业”高标准示范基地覆盖面积增加到50万亩以上，全市绿色有机食品基地面积稳定在800万亩左右。以“两牛一猪一禽”为重点，扶持双兆猪业、龙垦肉驴、盛世凯达等项目加快建设，推进畜牧业标准化、规模化、集约化生产，做好过腹增值文章。支持益海粮油、象屿金谷、金玛集团等龙头企业产业化扩张，延伸水稻、玉米、大豆等农副产品产业链，补齐就地转化和精深加工短板。健全完善农产品质量标准和追溯体系，发展农村电子商务、农超对接、农业众筹等新型农产品流通销售业态。精心打造“生态米都”城市新名片，充分发挥“佳木斯大米”品牌的引领作用，鼓励和支持龙头企业、专业合作社等主体开发符合网购需求的特色农产品，向青岛、杭州、深圳等大中城市拓展中高端市场，提升农产品价值链。加快现代农业经营体系建设。落实承包土地“三权分置”办法，促进适度规模经营。规范农民合作社100个以上，鼓励支持工商资本有序进入农业领域，嫁接改造传统农业。完善新型农业社会化服务体系，探索“政银保”合作模式，推进活体畜禽抵押贷款等保证、保险业务，开展农房、农机具险等创新试点。加强农业技能普及培训，培育新型职业农民。推动认养农业规模化发展、信息化建设。

提升工业实力。坚持传统产业和新兴产业并重，优化存量与引导增量并举，推进产业集聚，提高工业运行质量和效益，规模以上工业增加值增长3%左右。主动承接哈尔滨电气集团、约翰迪尔公司外委业务，拓展电机、农机产业市场空间。积极与小松公司接洽，争取煤机公司尽快启动生产并攻关盾构机等工程机械新产品。加快研发秸秆收获和处理机械，推动迪尔公司200马力以上大型稻麦联合收割机、邢台一拖水稻插秧机等新产品产业化，打造国家综合农机产业基地。推进润特科技高效膨胀型阻燃剂项目二期扩建和特超高压电缆项目建设，支持葵花药业化药新药转化基地落户我市，引导乌苏里江佳大制药等资产闲置企业招商重组，争取中国建材玻璃器皿等项目尽早开工。发挥“农机走出去”产业联盟平台作用，促进本地产业间、企业间融合发展。实施中小微企业梯次成长工程，引导其向专精特新发展转变、向规模以上企业扩张。构建符合县域特色的工业主导产业体系。

发展服务经济。重点培育物流、金融、电商、旅游等业态，释放新消费、创造新供给、形成新动力。构建物流体系。推进公铁多式联运物流园区、同江能源储运园区等项目。完善佳木斯电子商务产业园，拓展互联网、大数据等信息技术在服务领域的应用空间，促进电商同实体经济深度融合。优化金融服务。继续引进外阜银行在我市设立分支机构，引导企业上市融资。有效对接多层次资本市场，加快城市商业银行联合重组和农村商业银行、农村合作银行组建步伐。做强商贸流通。加快万达广场等城市综合体项目建设，启动运营0454等特色商业街区，有序发展高档酒店、连锁经营等业态。做实旅游产业。促进旅游与历史、民俗、冰雪、生态、文化等元素融合，构建“大旅游”格局。抓好重点景区基础设施完善、景区资源联动等工作，打造留得住游客的精品旅游线路。设计开发工业游园、农业景观等城市周边旅游产品，争取启动航空旅游市场。旅游收入增长10%以上。做大健康养老。推进光荣院民生养生养老中心等项目建设，全方位推动候鸟式养老产业发展。加强“医养融合”体系建设，鼓励医疗机构开展医养结合型的养老服务，打造区域养老产业特色品牌。做活地产开发。推出差异化的住宅产品，在引导房地产业差别竞争、健康发展的同时，消化现有库存，有效释放刚性需求和改善性需求。

推动创新创业。强化企业创新主体地位，推动科技同经济对接、创新成果同产业对接。坚持引进消化吸收再创新，重点推进电机股份高温气冷堆核电站示范工程核反应堆项目加快转化，迪尔制药全自动扣壳机、汤原海瑞特聚芳酯合成等一批项目产业化生产。新建生物质应用和食用菌产业技术创新战略联盟，新注册成立科技型企业120户，新增高新技术企业5户，高新技术产业增加值同比增长8%以上。全面提升现有14个科技孵化器的服务功能，充分发挥佳木斯大学科技园等创业园区作用，新增创新创业基地5个，营造靠创业自立、凭创新出彩的浓厚社会氛围。

（三）深入推进改革开放。最大限度释放改革红利，拓展开放空间。

加大“放管服”等重点领域和关键环节的改革力度，营造规则优势、政策优势和服务优势。继续深化行政审批制度改革，动态调整“三张清单”，优化审批及公共服务流程，提高集中审批和便民服务事项网上审批率。探索建立政府收费清单及行政事业收费清单。加快公共服务管理标准化建设，扩大公共资源市场化配置领域和范围，推动“两随机、一公开”监管方式全覆盖。用好“政策工具箱”和“智慧工具箱”，压降企业“时间成本”“财务成本”“生产成本”，用心用情构建“亲”“清”新型政商关系。深化财税体制改革。全面实施“营改增”，按照新体制要求组织财政收入，优化税收征管环境。创新财政支出方式，注重发挥专业咨询机构作用，在基础设施和公用事业领域采取PPP等方式，引导社会资本参与重点项目建设，增强政府再投资和公共产品供给能力。落实地方政府金融监管责任，防范和化解金融风险。统筹推进“三权合一”事业单位转企改制等项改革。

积极融入国家“一带一路”和“龙江陆海丝路带”总体布局，加快完善跨境产业链条。推动同江中俄跨江铁路大桥俄方建设，争取开通我方至俄萨哈林江海联运航线和佳木斯至首尔、哈巴等国际货运航线。大力推进佳木斯综合保税区和同江跨境经济合作区建设，扩大佳天国际海关监管库对俄进出口规模。利用俄方超前发展区的优惠政策，谋划推进哈巴罗夫斯克佳木斯工业园、中铁龙兴阿穆尔园等境外园区建设。寻求与阿里巴巴速卖通等跨境电商合作，建设佳木斯跨境电子商务平台。启动国储集团（佳木斯）投资有限公司合作项目，加大对桦南恒源、同江祥龙等一批有规模和潜力创汇型企业的扶持力度，不断优化贸易结构，拓展欧洲、美洲等国际市场，外贸进出口总额增长3%左右。广泛开展教育、文化、旅游、卫生、科技等方面的国际交流互访活动，以人文交流促进经济合作。

（四）扎实开展脱贫攻坚。全面落实国务院《“十三五”脱贫攻坚规划》，集中力量攻克薄弱环节，确保完成57个贫困村、5.5万贫困人口的年度脱贫任务。

持续抓好建档立卡动态调整，强化精准识别精准施策，确保应扶尽扶。扎实推进教育、医疗、社会力量和社会保障兜底扶贫等措施，实行因户施策和资源的最优配置，制定个性化的扶贫方案，精准落实责任。发挥政府主导和市场引导作用，推广“一村一品”带动、新型农业经营主体带动、旅游开发带动等模式，实现产业与贫困户有效对接，促进扶贫开发由“输血”向“造血”转变。严格贫困退出的办法和程序，建立防止返贫机制，做到贫困问题随时产生、随时消除，切实巩固脱贫成果。开工建设北京至抚远公路佳木斯至桦川段等四条国省级道路，升级改造县级公路路网。改造农村危（草）房9000户，解决30个村屯2.4万人口饮水安全问题。加强农村基础设施和人居环境建设，打造美丽乡村100个以上。

（五）持续增进民生福祉。顺应民意，倾力作为，让城市的发展更有“温度”，让群众的幸福更有“质感”。

推动市、县、乡三级创业载体完善功能、提档升级，为创业者提供高效便捷服务，新增城镇就业4.5万人以上，城镇登记失业率控制在4.5%以内，零就业家庭动态清零。整合城乡居民基本医疗保险制度，确保150万名城乡居民享受同等医保待遇。按照不低于上年度城乡人均消费支出30%和80%的水平，合理提高城乡低保和特困人员救助供养标准。发展多层次社会化养老服务，建设服务面积300平方米以上的标准化城市社区居家养老服务中心10家。提高城市规划的前瞻性、科学性，推行“多规合一”，强化执行刚性。推进城市“双修”，促进城市发展模式和治理方式转型。加快学府街公铁立交桥、胜利路公铁立交桥、浦东街南段等16项路桥建设工程，打通浦东街南段、育林路等6条断头路。美化安庆、万兴等五个城市出入口，打造迎宾绿化景观。规划建设华融大厦、银鼎明苑、北苑花园、商业城南门等4个停车场，新建改建公厕15座。开展“五整治五规范”，提升城市管理长效化、科学化、精细化水平。优先发展公共交通，更新纯电动公交车35辆，新建公交站亭50个。完成天福热源建设，市区新增供热能力200万平方米；改造老旧供热管网30公里，实现集中供热管网联通，应急状态下热能可调控互补；淘汰10蒸吨及以下燃煤锅炉50台；新建、改造燃气管网38公里，安装燃气用户1万户。启动江北水源地迁建，实施城区水质升级改造工程。扩建格节河水库，建设城市应急备用水源。推进“海绵城市”建设，建成地下管廊5.74公里。新开工市本级棚户区改造项目11000套，分配公共租赁住房400套，新增廉租房租金补贴700户；规范住宅小区物业管理，维修老旧小区楼房120栋。落实中央环保督查组整改要求，推进大气、水和土壤污染防治行动计划，松花江干流佳木斯段III类水质和环境空气质量优良天数比例分别达90%以上。保护湿地资源，造林绿化8.6万亩。按照“一县（市）一主题、一镇一风格”的总体思路，做好城关镇、重点中心镇、特色小城镇建设，推动基本公共服务向镇村延伸。落实学前教育三年行动计划,学前教育三年毛入园率达85%；推动“三通两平台”工程，“校校通”完成率达100%，“班班通”城镇学校完成率达100%，农村学校完成率达85%。加快教育现代化建设，推动优质教育资源均衡发展。适应产业转型和创业就业需求，合理设置职业教育专业，建设一批有示范性的实训基地。稳妥推动城市公立医院改革；新建10个自助式标准化健康小屋，方便社区居民进行高血压、糖尿病等疾病筛查；实现二、三级医院新农合住院患者基本医疗保障、大病商业保险即时结算；全面推进“健康佳木斯”建设，开展健康巡讲100场以上。坚持和完善计划生育制度。在33个贫困村、34个示范村建设综合性文化活动室（中心）和文化广场，推进市级“三馆”和县级“两馆”达标建设。加快公共体育场馆市场化运营，利用“互联网＋”等手段提高场馆便民利用率。以社会主义核心价值观提升塑造城市文明，营造向上向善的社会秩序和社会氛围。新建办公面积300平方米以上的标准化城市社区20个，试行政府向符合条件的社会组织和专业社工购买服务。强化食品药品安全社会共治责任，开展“药品安全放心年”活动，争创国家食品安全城市。赫哲族在全国28个人口较少民族中率先实现全面小康。

（六）保持社会和谐稳定。层层落实责任，强化综合治理，为党的十九大胜利召开营造良好的社会环境。

坚守“发展决不能以牺牲安全为代价”这条不可逾越的红线，落实好“党政同责、一岗双责、齐抓共管、失职追责”的安全生产责任体系，建立公共安全源头管理机制，实行重大安全风险“一票否决”。打造阳光信访、法制信访、责任信访，完善社会矛盾排查预警和调处化解综合机制，重点解决好动迁户回迁、房屋无法办照等群众高度关注的信访积案，切实维护群众的合法利益、合理诉求。以信息化为支撑，完善立体化社会治安防控体系，推进“平安佳木斯”建设，保障人民群众安居乐业。完善相关预案，增强处置能力，有效应对突发事件，防范社会风险。落实“七五”普法规划，加快“法治佳木斯”建设进程。

三、切实加强政府自身建设

认识新常态、适应新常态、引领新常态，对政府工作提出了更高要求。我们要与时俱进转变政府职能，驰而不息优化发展环境，进一步增强政府的执行力和公信力。

（一）提升创新干事本领。全面深入持续地学习贯彻习近平总书记系列重要讲话精神，长效化推进“两学一做”学习教育和专项业务培训，在理念创新中引领工作创新，培养政府工作人员的系统思维、专业能力和市场经济意识，勤于修炼能干会干的“过硬内功”，努力锻造担当尽责的“看家本领”，带头讲法治、讲规则、讲诚信。坚持问题导向，破除惯性思维，以开放的胸襟对待新生事物，以包容的心态回应社会关切，积极运用市场机制、经济手段、法治办法推动发展，使政府有形之手、市场无形之手、市民勤劳之手同向发力。

（二）锤炼务实为民作风。坚持“民之所望、施政所向”，让目标制定和工作推进的过程，成为集中民智、凝聚民力的过程。把“严”的作风和“实”的态度融入政府日常工作，靠勇于担当破解千难万难，靠主动作为闯出大路小路，以敬业精神、专业精神和工匠精神推动工作好中求快、优中求进。完善督办检查机制，严肃行政监察问责，大力整治不作为、慢作为、虚作为、乱作为。系统落实《黑龙江省法治政府建设实施方案》（2016-2020年），按照法定权限和程序行使权力，主动接受人大及其常委会法律和工作监督、政协民主监督、社会舆论监督。继续深化政务公开，推进决策、执行、管理、服务、结果“五公开”。加强政府智库建设。

（三）坚持廉洁自律操守。从严落实中央八项规定精神，压实全面从严治党的主体责任，把纪律规矩挺在法律前面，让监督执纪“四种形态”成为全面从严治党常态，切实维护《准则》《条例》的权威性。严格执行四个“一律不许”，严肃查处各类违法违纪案件，维护良好的政治生态和从政环境，在干净做事中推动发展、成就事业、服务社会，不断增强人民群众对政府的信心、信任和信赖。

各位代表，站在新的起点，创造新的业绩，是时代的召唤、人民的重托，是历史赋予我们的光荣使命和神圣职责。让我们在市委的坚强领导下，真抓实干、担当有为，攻坚克难、砥砺前行，为全面建成小康社会而努力奋斗！
